# Socioeconomic determinants of growth in a longitudinal study in Nepal

**DOI:** 10.1111/mcn.12462

**Published:** 2017-04-27

**Authors:** Delan Devakumar, Dalvir Kular, Bhim P. Shrestha, Carlos Grijalva‐Eternod, Rhian M. Daniel, Naomi M. Saville, Dharma S. Manandhar, Anthony Costello, David Osrin, Jonathan C.K. Wells

**Affiliations:** ^1^ Institute for Global Health, UCL London UK; ^2^ Mother and Infant Research Activities Kathmandu Nepal; ^3^ London School of Hygiene and Tropical Medicine London UK; ^4^ Nutrition Unit, GOS Institute of Child Health, UCL London UK

**Keywords:** asset index, body composition, child growth, land ownership, maternal education, socioeconomic factors

## Abstract

Socioeconomic status (SES) is associated with childhood anthropometry, but little is known about how it is associated with tissue growth and body composition. To investigate this, we looked at components of SES at birth with growth in early and mid‐childhood, and body composition in a longitudinal study in Nepal. The exposure variables (material assets, land ownership, and maternal education) were quantified from questionnaire data before birth. Anthropometry data at birth, 2.5 and 8.5 years, were normalized using WHO reference ranges and conditional growth calculated. Associations with child growth and body composition were explored using multiple regression analysis. Complete anthropometry data were available for 793 children. There was a positive association between SES and height‐for‐age and weight‐for‐age, and a reduction in odds of stunting and underweight for each increase in rank of SES variable. Associations tended to be significant when moving from the lower to the upper asset score, from none to secondary education, and no land to >30 dhur (~500 m^2^). The strongest associations were for maternal secondary education, showing an increase of 0.6–0.7 *z* scores in height‐for‐age and weight‐for‐age at 2.5 and 8.5 years and 0.3 kg/m^2^ in fat and lean mass compared to no education. There was a positive association with conditional growth in the highest asset score group and secondary maternal education, and generally no association with land ownership. Our results show that SES at birth is important for the growth of children, with a greater association with fat mass. The greatest influence was maternal secondary education.

**Abbreviations**
CH: conditional height (CH)
CRW: conditional relative weight
HAZ: height‐for‐age z‐score
SES: socioeconomic status
WAZ: weight‐for‐age z‐score

## 1 INTRODUCTION

Socioeconomic status (SES) is associated with nutritional status in children in low‐ and middle‐income countries (Barros et al., 2006; Fernald, Kariger, Hidrobo, & Gertler, 2012; Menezes et al., 2011; Meshram et al., 2012), but its associations with somatic growth patterns in different periods of childhood is less well understood.

SES is a complex concept that incorporates social status (including social position, agency, and capital) and wealth (Conger & Donnellan, 2007). It is a distal factor believed to act on phenotype through a number of proximal pathways. Lower SES has been found to be associated with worse health outcomes in childhood (Spencer, Thanh, & Louise, 2013), lower birth weight (Blumenshine, Egerter, Barclay, Cubbin, & Braveman, 2010; Martorell & Zongrone, 2012), and child mortality (Cleland, Bicego, & Fegan, 1992; Houweling & Kunst, 2010). In addition, a lower SES early in life has been associated with a lower attained adult height (Black et al., 2008), lower educational achievement, and reduced income (Fernald et al., 2012), all of which may help perpetuate intergenerational cycles of malnutrition and poverty (Conger & Donnellan, 2007; Victora et al., 2008).

Evidence is also emerging of the importance of child growth (i.e., a change in anthropometric indices over time) during different periods in childhood, not only for immediate health but also for longer term health and education (Adair et al., 2013). In addition, child growth is an important short‐ and long‐term marker and predictor of illness. The mechanisms by which SES might act upon child growth are known to be complex. For instance, it might alter illness patterns at different time points; such that a low SES may not only first increase the risk of contracting an illness, perhaps via poor sanitation, but may also later reduce the ability to treat such illness, perhaps via the family's limited capacity to access and afford healthcare. SES influence on the risk and severity of illness can in turn increase the metabolic demands on a growing child and reduce their ability to absorb nutrients, thus impairing growth. A greater understanding of how and when SES affects growth is important, particularly in countries in which poverty and undernutrition are common.

Much of our understanding about the relationship between SES and nutritional indicators is that index child growth comes from cross‐sectional studies, such as Demographic and Health Surveys, resulting in difficulty in inferring causality. In addition to this, the available evidence tends to report measures of anthropometry at single time points, with minimal body composition data, and do not include SES exposure prior to birth.

Using data from a longitudinal study in the south of Nepal, we aimed to examine the potential associations with child growth outcomes of three different distal components of SES, namely, household material assets, maternal education, and land ownership, all measured before birth. The outcomes comprised child anthropometry at three time points, growth in early childhood (0–2.5 years) and mid‐childhood (2.5–8 years), and body composition at 8.5 years.

**Key messages**

- Socioeconomic components at birth determine childhood anthropometry, conditional growth, and body composition.
- Maternal secondary education showed the strongest association with child growth and body composition.
- SES associations with body mass at 8.5 years were mostly attributable to fat mass variability.

## 2 PARTICIPANTS AND METHODS

### 2.1 Setting

Nepal is among the poorest countries in the world. Its gross domestic product is estimated to be US$19.4 billion in real terms or $40.0 billion (US$1,500 per capita) in purchasing power parity, ranking it 102nd in the world (Central Intelligence Agency, 2013; Klugman, 2011). The 2011 Demographic and Health Survey estimated that 29% of children under five were underweight, 41% were stunted, and 11% were wasted (Ministry of Health and Population [Nepal] New ERA & Inc I.I, 2012). The study was conducted in and around the city of Janakpur, Dhanusha, in the Central Terai region. Dhanusha is situated in the south‐eastern region of Nepal on the border with India. It covers an area of 1180 km^2^ and, with a population of 754,777, is the fifth most populous state in Nepal. Janakpur is the district capital and has a population of 98,446 (Central Bureau of Statisics, 2012). The previous study on which the analysis is based showed that 18%, 37%, and 53% of children were underweight (weight‐for‐age ≤ 2 *z* scores) at birth, 2.5 and 8.5 years, respectively; 9%, 59%, and 29% were stunted (height‐for‐age ≤ 2 z scores) at birth, 2.5 and 8.5 years, respectively (Devakumar et al., 2014).

### 2.2 Procedures

The participants were children followed up from a randomised controlled trial of antenatal multiple micronutrient supplementation. The full details of the trial have been described elsewhere. Briefly, the sampling frame for potential participants included all women attending Janakpur Zonal Hospital for antenatal appointments prior to 20 weeks of gestation. This included women who live in both urban and rural locations. Infants were then assessed in the hospital or at home within 72 hr of birth. Exclusions were multiple pregnancies, foetal abnormalities, and maternal illness of a severity that could compromise the outcome of pregnancy and if they lived outside of the region of Dhanusha and the adjoining region of Mahottari; 30% of mothers were <20 years old, 65% between 20 and 29 years old, and 5% ≥30 years old. Fifty‐three percent of participants were urban dwelling (Osrin et al., 2005).

Antenatal multiple micronutrient supplementation appeared to have no effect on length/height, no lasting effect on weight at 8.5 years of age, and no effect on conditional relative growth, so all children from intervention and control groups were included in the current study (Devakumar et al., 2014). The children born in the trial were seen within 72 hr of birth and again at a mean 2.5 and 8.5 years of age. Assessments at 2.5 and 8.5 years were performed in a central research site in Janakpur by trained data collection staff (Vaidya et al., 2008; Devakumar et al., 2014). Measurements of height and weight were taken at each time point. Mean length/height was estimated from duplicate measures taken at birth, using a Kiddimetre board or Rollametre accurate to 1 mm (Raven Equipment, Castlemead, UK), and at ages 2 and 8 years, using a Leicester stadiometer accurate to 1 mm. The children were barefoot and placed in the standard position, laying on the floor with head and legs held in position at birth, and upright with knees extended, feet together, and head in the Frankfort plane at later follow‐ups. Sitting height was measured with the child seated on a custom‐made stool with the base of the spine touching the stadiometer and head in the Frankfort plane. Weight was measured at birth and 2 years, using Seca 835 electronic scales (Hamburg, Germany) appropriate for infants and young children and accurate to 10 g, and at 8 years using Tanita BC‐418 scales (Tanita Corp, Japan) accurate to 100 g. Scales were tared before each measurement and calibrated regularly. Body composition was assessed only at 8 years, using bioelectrical impedance with locally derived prediction equations obtained using isotope calibration (Devakumar et al., 2015).

Information regarding our proxies for SES was gathered using questionnaire data collected at birth. The questions included an asset rank recommended at the time by the World Health Organization that stratified households into four categories, with more expensive items like a motor vehicle or a refrigerator given the highest ranking and having none of the items as the lowest rank (WHO, 1994). The highest education level of the mother was recorded, and the amount of land owned by the family was estimated.

The current follow‐up was approved by the University College London Research Ethics Committee (Ethics Project ID 2744/001) and the Nepal Health Research Council (Reference 51/2011).

### 2.3 Statistical analysis

WHO Child Growth Standards for children under five (WHO Multi‐Centre Growth Reference Study Group, 2009) and aged 5–19 (de Onis et al., 2007), were applied to create *z* scores for weight‐for‐age (WAZ) and height‐for‐age (HAZ), adjusted for sex. We defined stunting and underweight as HAZ and WAZ < −2 *z* scores, respectively. Longitudinal child growth was defined as conditional height (CH) and conditional relative weight (CRW) at two periods, 0–2.5 and 2.5–8.5 years, using the method described by Adair et al. (2013). This calculates the change in growth from that expected for the child on the basis of previous size measures and accounts for the regression towards the mean, a known problem when assessing longitudinal growth (Adair et al., 2013). A positive value represents faster than expected growth. CH takes into account previous weight and height values (as *z* scores), whereas CRW accounts for previous weight and height, and current height values (calculated as standardised residuals). CH can be considered a marker of skeletal growth and CRW a marker of soft tissue growth. The inclusion of birth data in the 8.5‐year relative growth analysis had no effect on the model.

Relative leg length at 8.5 years of age was calculated as the ratio of leg length to height, presented as a percentage. Body mass index (BMI) at this age was calculated as weight (kg) divided by squared height (metres). Lean mass index (LMI) and fat mass index (FMI) were calculated as in Equation (1).

(1) $$\begin{array}{l} \mathrm{BMI}=\mathrm{FMI}+\mathrm{LMI}\\ \frac{\text{Weight}}{{\text{Height}}^{2}}=\frac{\mathrm{Fat}\;\text{mass}}{{\text{Height}}^{2}}+\frac{\text{Lean}\;\text{mass}}{{\text{Height}}^{2}} \end{array}$$

Asset score, maternal education, and land ownership at birth were included as ordinal data. The two middle categories of asset score were merged due to low numbers, resulting in three levels (WHO, 1994). Land ownership was classified as no land, ≤30 dhur (~500 m^2^), and >30 dhur. Maternal education was classified as no education, primary, and secondary or higher education. Categories of SES variables are referred to in Tables 2–4 as levels 1 to 3 denoting their ascending order.

We investigated the association between SES and child growth using multiple regression, controlling for the antenatal trial allocation. No other confounders were included in the models as the socioeconomic variables were the most distal in our conceptual model of putative relationships. We studied the association between SES and child growth in three different ways. First, we used linear regression to assess separately the relationship between each of asset score, maternal education, and land ownership, as exposure variables, and anthropometric indices HAZ, WAZ, or body composition variables, BMI, fat mass index, and lean mass index, as outcome variables. Second, we used logistic regression to estimate the odds of stunting or underweight for each of the same exposure variables. Lastly, we used linear regression to assess the association between CH and CRW and each of the exposure variables. Statistical analysis was done in Stata (version 12.1; Stata Corp, USA).

## 3 RESULTS

**Figure 1:**
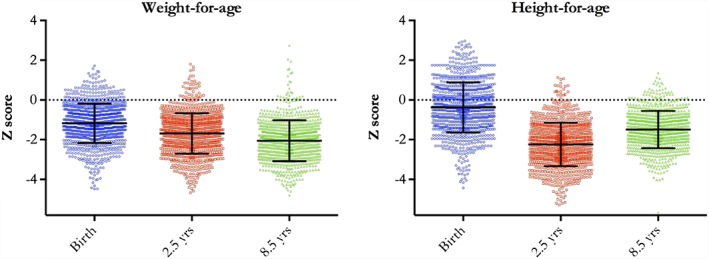
Change in *z* scores. Black bars denote mean ± 1 standard deviation

Table 1 shows characteristics of the cohort. Complete anthropometry data were available for 793 children (48.3% of whom were girls), representing 75.3% of available children (excluding deaths) whose mothers completed the original trial. Body composition data were collected from 628 children. Children lost to follow‐up more likely to be urban residents (*p* < .0001) and their mothers had some education (*p* < .0001) but were otherwise similar to those retained in the study. The mean ages at follow‐up at the three time points were 0 (<72 hr of age), 2.5, and 8.5 years. At 8.5 years of age, just over half the children were underweight and approximately one third were stunted. Figure 1 shows scatterplots for HAZ and WAZ from birth to 8 years. For HAZ and WAZ, we observed an average reduction of 1.15 and 0.87 *z* scores between birth and 8 years of age, respectively. Compared to HAZ, WAZ reduction was lower in this period, but infants were relatively lighter at birth. For HAZ, there appeared to be some recovery between 2.5 and 8.5 years of age, whereas WAZ showed a steady decline.

**Table 1:** Characteristics of cohort at enrolment *30 dhur = approximately 500 m^2^

	Frequency (%)
Child sex	
Female	383 (48.3)
Male	410 (51.7)
Maternal education	
None	401 (50.1)
Primary	67 (8.4)
Secondary or higher	325 (41.0)
Main household livelihood	
No work	88 (11.1)
Some work, either self‐employed or employed	705 (88.9)
Land ownership	
0	84 (10.6)
<30 dhur	550 (69.4)
>30 dhur	159 (20.1)
Asset score	
None of the list assets (level 1)	120 (15.1)
Sewing machine, cassette player, camera, fan, bullock cart, wall clock, radio, iron, bicycle (level 2)	264 (33.3)
Motor vehicle, TV, or refrigerator (level 3)	413 (51.6)

### 3.1 Associations between SES and child linear growth (Table 2)

The most consistent pattern of associations was between maternal education and HAZ, where each level increase of maternal education showed a positive increase on HAZ values at all ages. A similar but more attenuated pattern was observed for assets score, where positive and significant increases for HAZ values were observed for all levels at 2.5 years of age and only at the higher level at 8.5 years of age. Land ownership showed a less clear pattern, where only high levels of land ownership showed a positive increase on HAZ.

**Table 2:** Association between SES indicators and growth in height

Regression coefficients for HAZ												
	Assets score				Maternal education				Land ownership			
	Level 2		Level 3		Level 2		Level 3		Level 2		Level 3	
	β	95% CI	β	95% CI	β	95% CI	β	95% CI	β	95% CI	β	95% CI
Birth	0.10	[−0.17, 0.38]	0.17	[−0.10, 0.43]	0.07	[−0.26, 0.40]	0.21	[0.02, 0.39]	−0.01	[−0.29, 0.28]	0.17	[−0.17, 0.50]
2.5 years	0.24	[0.01, 0.46]	0.62	[0.41, 0.83]	0.27	[0.00, 0.53]	0.75	[0.60, 0.90]	0.11	[−0.13, 0.36]	0.38	[0.10, 0.66]
8.5 years	0.19	[−0.01, 0.38]	0.56	[0.38, 0.74]	0.28	[0.05, 0.51]	0.59	[0.46, 0.72]	0.06	[−0.15, 0.27]	0.31	[0.07, 0.55]
Odds ratio for stunting [HAZ < −2]												

	Assets score				Maternal education				Land ownership			
	Level 2		Level 3		Level 2		Level 3		Level 2		Level 3	
	OR	95% CI	OR	95% CI	OR	95% CI	OR	95% CI	OR	95% CI	OR	95% CI
Birth	0.88	[0.44, 1.77]	0.77	[0.40, 1.51]	0.62	[0.24, 1.61]	0.60	[0.35, 1.00]	0.72	[0.36, 1.44]	0.53	[0.22, 1.26]
2.5 years	0.56	[0.35, 0.89]	0.31	[0.20, 0.49]	0.56	[0.34, 0.94]	0.29	[0.21, 0.39]	0.95	[0.61, 1.49]	0.57	[0.34, 0.95]
8.5 years	0.83	[0.54, 1.27]	0.43	[0.28, 0.66]	0.59	[0.33, 1.04]	0.45	[0.32, 0.62]	1.04	[0.64, 1.68]	0.77	[0.43, 1.36]
Standardised regression coeffiCIents for conditional growth in height												

	Assets score				Maternal education				Land ownership			
	Level 2		Level 3		Level 2		Level 3		Level 2		Level 3	
	β	95% CI	β	95% CI	β	95% CI	β	95% CI	β	95% CI	β	95% CI
0–2.5 years	0.14	[−0.07, 0.35]	0.49	[0.30, 0.69]	0.21	[−0.04, 0.45]	0.65	[0.51, 0.78]	0.13	[−0.09, 0.36]	0.36	[0.10, 0.62]
2.5–8.5 years	0.16	[−0.05, 0.38]	0.32	[0.11, 0.52]	0.03	[−0.23, 0.29]	0.18	[0.04, 0.33]	−0.04	[−0.27, 0.19]	0.07	[−0.19, 0.34]

*Note*. β = regression coefficient; CI = confidence interval; HAZ = height‐for‐age *z* score; OR = odds ratio; SES = socioeconomic status.

The comparator group for the SES variables are asset level 1, no maternal education, or no land.

A similar pattern of associations was observed with the odds of being stunted at different ages. Maternal education showed the most consistent protective effect against stunting at all ages. Greater assets score levels at birth were protective against stunting mainly at 2.5 years but also at 8.5 years for the higher level of assets score.

Only the higher levels of all three SES proxies showed a positive increase in conditional height growth between 0 and 2.5 years of age, and only the higher levels of maternal education and assets score between 2.5–8.5 years of age.

### 3.2 Associations between SES and growth in weight (Table 3)

Maternal education showed the most consistent association with WAZ. Each level increase of maternal education showed a significant increment of WAZ at all ages, except at birth when comparing primary education versus no education. Again, a similar pattern was seen with assets score but only the higher levels of land ownership showed a positive increase in WAZ at 2.5 and 8.5 years.

**Table 3:** Association between SES indicators and growth in weight

Regression coefficients for WAZ												
	Assets score				Maternal education				Land ownership			
	Level 2		Level 3		Level 2		Level 3		Level 2		Level 3	
	β	95% CI	β	95% CI	β	95% CI	β	95% CI	β	95% CI	β	95% CI
Birth	0.24	[0.04, 0.45]	0.27	[0.08, 0.47]	0.16	[−0.09, 0.40]	0.21	[0.08, 0.36]	−0.07	[−0.29, 0.14]	−0.03	[−0.28, 0.22]
2.5 years	0.18	[−0.04, 0.39]	0.47	[0.27, 0.67]	0.38	[0.14, 0.63]	0.63	[0.49, 0.77]	0.04	[−0.18, 0.27]	0.34	[0.08, 0.60]
8.5 years	0.20	[−0.01, 0.41]	0.63	[0.43, 0.83]	0.29	[0.05, 0.54]	0.70	[0.56, 0.84]	−0.03	[−0.25, 0.20]	0.31	[0.05, 0.58]
Odds ratio for underweight [WAZ < −2]												

	Assets score				Maternal education				Land ownership			
	Level 2		Level 3		Level 2		Level 3		Level 2		Level 3	
	OR	95% CI	OR	95% CI	OR	95% CI	OR	95% CI	OR	95% CI	OR	95% CI
Birth	0.72	[0.43, 2.00]	0.65	[0.40, 1.06]	0.86	[0.45, 1.64]	0.65	[0.44, 0.95]	1.75	[0.90, 3.41]	1.38	[0.65, 2.93]
2.5 years	0.76	[0.50, 1.16]	0.50	[0.33, 0.74]	0.61	[0.36, 1.02]	0.33	[0.24, 0.45]	0.97	[0.62, 1.53]	0.70	[0.41, 1.18]
8.5 years	0.77	[0.50, 1.19]	0.39	[0.26, 0.59]	0.54	[0.32, 0.89]	0.28	[0.21, 0.39]	1.09	[0.70, 1.69]	0.68	[0.41, 1.13]
Standardised regression coefficients for conditional growth in weight												

	Assets score				Maternal education				Land ownership			
	Level 2		Level 3		Level 2		Level 3		Level 2		Level 3	
	β	95% CI	β	95% CI	β	95% CI	β	95% CI	β	95% CI	β	95% CI
0–2.5 years	0.00	[−0.21, 0.21]	0.08	[−0.12, 0.28]	0.28	[0.03, 0.54]	0.21	[0.06, 0.35]	0.03	[−0.26, 0.20]	0.15	[−0.11, 0.42]
2.5–8.5 years	0.06	[−0.16, 0.27]	0.21	[0.02, 0.42]	−0.11	[−0.37, 0.15]	0.22	[0.07, 0.36]	−0.14	[−0.37, 0.08]	−0.03	[−0.29, 0.24]

*Note*. β = regression coefficient; CI = confidence interval; WAZ = weight‐for‐age *z* score; OR = odds ratio; SES = socioeconomic status.

The comparator group for the SES variables are asset level 1, no maternal education, or no land.

For the odds of being underweight, we observed that at most ages, only the higher levels of maternal education or assets score showed a protective effect against being underweight in children. Greater levels of land ownership before birth did not show a protective effect against being underweight.

The higher levels of maternal education had a significant positive association with greater conditional growth in weight on both periods, 0–2.5 and 2.5–8.5 years.

### 3.3 Associations between SES and body proportions and composition at 8.5 years (Table 4)

The SES proxies did not show a significant effect on relative leg length. Only the greater level of each SES proxy showed a positive increase in BMI values at 8.5 years. When compartmentalising that positive effect into its two components, fat and lean mass, all three SES proxies showed a significant positive increase of fat mass, but only maternal education showed a significant positive increase of lean mass.

**Table 4:** Association between SES indicators and body composition and proportions at 8.5 years of age

Regression coefficients for body composition indices and relative leg‐length												
	Assets score^1^				Maternal education^2^				Land ownership^3^			
	Level 2		Level 3		Level 2		Level 3		Level 2		Level 3	
	β	95% CI	β	95% CI	β	95% CI	β	95% CI	β	95% CI	β	95% CI
Leg length	−0.06	[−0.30, 0.18]	0.12	[−0.10, 0.35]	−0.01	[−0.30, 0.28]	0.16	[−0.00, 0.32]	0.15	[−0.10, 0.40]	0.23	[−0.06, 0.52]
BMI	0.16	[−0.11, 0.43]	0.52	[0.26, 0.78]	0.18	[−0.15, 0.51]	0.58	[0.40, 0.77]	−0.09	[−0.37, 0.20]	0.26	[−0.07, 0.60]
LMI	0.08	[−0.16, 0.33]	0.18	[−0.06, 0.41]	0.13	[−0.15, 0.41]	0.26	[0.11, 0.42]	−0.25	[−0.52, 0.01]	−0.07	[−0.38, 0.23]
FMI	‐0.01	[−0.24, 0.23]	0.25	[0.03, 0.48]	0.12	[−0.15, 0.38]	0.33	[0.18, 0.48]	0.17	[−0.09, 0.42]	0.40	[0.11, 0.69]

*Note*. β = regression coefficient; BMI = body mass index; FMI = fat mass index; LMI = lean mass index; SES = socioeconomic status.

The comparator group for the SES variables are asset level 1, no maternal education, or no land.

## 4 DISCUSSION

Our results are consistent with the findings of others, namely, that the family's SES collected at birth is an important factor affecting the anthropometric status of children. Generally, all SES proxies showed a positive association with HAZ and WAZ at 2.5 and 8.5 years, with mixed results at birth. Most of the associations were in the same direction, from the poorest to the intermediary to the least poor level. However, not all SES proxies had the same pattern of associations with the different types of child growth. We observed the most consistent pattern of associations for maternal education, whereas land ownership showed the least clear pattern. We have shown that SES is associated with the growth of skeletal tissue (HAZ and conditional growth in height) and of soft tissue (WAZ and conditional relative growth in weight). Furthermore, our results indicate that SES associations with body mass at 8.5 years were mostly attributable to fat mass variability, though maternal education was also associated with lean mass.

Children in our sample started life, on average, underweight and a little shorter than reference children of similar age and sex (de Onis et al., 2007; WHO Multi‐Centre Growth Reference Study Group, 2009). Both height and weight reduced relatively over childhood. For height, this was greatest in the earlier period.

Generally, SES proxies did not show a protective effect against being stunted or underweight at birth, but its protective effects were seen later in life. Interestingly, the protective effects were seen more clearly against stunting than against underweight. As expected, the most consistent pattern of associations between SES proxies and child growth and body composition was seen when moving from the lowest to the highest level of SES. Maternal secondary education appeared to have the strongest effect and was the only exposure that affected lean mass. In our results, changes in fat mass were more amenable to changes in SES.

Our definition of growth was not just a change in height or weight, but a deviation from expected values. The associations were generally greater for both conditional height and weight gain in the early life period, as might be expected as socioeconomic factors before birth will wear off over time.

The supporting information summarises the evidence from longitudinal studies that have investigated the association between the socioeconomic marker as in our study, and growth. Positive associations were seen between asset ownership and child growth in Ethiopia, India, Peru, Vietnam (Krishna et al., 2015), and the Philippines (but not South Africa in the same study; Jones et al., 2008), but this was not always consistent. Lourenço et al. considered children of a similar age to ours and showed a positive association between wealth and height (Lourenco, Villamor, Augusto, & Cardoso, 2012).

Maternal education was the strongest explanatory variable, particularly for skeletal growth in early childhood. Cross‐sectional studies across multiple countries have shown a positive association of maternal education with offspring height, or a negative association with stunting (Lakshman et al., 2013; Moestue & Huttly, 2008; Semba et al., 2008; Tiwari, Ausman, & Agho, 2014). Similar findings on the importance of maternal education, particularly in early life, have been described in a longitudinal study in Brazil. Matijasevich et al. showed that maternal education was positively associated with differences in growth rates in height/length < 2.5 years of age (though not from 3 to 12 months) and that there was a lack of association later in childhood up to 4 years (Matijasevich et al., 2012). Busert et al. (2016) did not find an association with height for age difference in a mountainous region of Nepal; however, childhood stunting has been shown to be more common when mothers have no education than when they complete school or higher education in Nepal (Ministry of Health and Population [Nepal] et al., 2012). How maternal education affects growth is complex, and may be mediated by attitudes, beliefs, and care practices that follow from education (Wachs, 2008). One example is through health‐seeking behaviour. For example, mothers with secondary education have been found to be more likely than those with no education to take children with diarrhoea to a health facility or provider (50% vs. 34%; Ministry of Health and Population [Nepal] et al., 2012). The effect of maternal education during early growth may be due in part to its proximal impact on infant and young child‐feeding practices that are known to impact anthropometric outcomes (Lamichhane et al., 2016). A cross‐sectional study in Nepal found that mothers with secondary or higher education were more likely to introduce complementary foods on time (adjusted OR 2.10; 95% CI 1.01–3.94) and to provide a minimum acceptable diet in the last 24 hr, OR 2.15; 95% CI [1.02, 4.54] (Gautam, Adhikari, Khatri, & Devkota, 2016). Our work adds further evidence to calls for improvement in maternal education.

The importance of land ownership varies by location and from family to family. The association with growth varies according to geography, climate, type of land, and the dependence on land for income or food. There is a paucity of studies examining the relationship between the distal factor of land ownership and child growth. Lourenco et al. found a positive association between land ownership and child growth; however, this was only in later child growth and not early growth (Lourenco et al., 2012). Cross‐sectional studies in Bangladesh showed that ownership of cultivable land was found to be protective against poor dietary diversity (Rah et al., 2010) and reduced incidence of diarrhoea and better weight and height gains compared to less land or landless (Hussain & Smith, 1999; Torres et al., 2000).

### 4.1 Limitations

We acknowledge that SES is a complex concept to define and that there are many alternative methods (Howe et al., 2012). We used three variables as proxies, each of which has limitations, but we feel that they cover different aspects of SES. We did not have accurate measures of consumption and felt that income would be unreliable as in this setting it is often seasonal, may be nonmonetary and may not be reported accurately. Our measure of land did not capture its location and importance to the family. A further limitation of the study was that many of the original participants chose to give birth at the main public hospital. These women were not from the poorest families (who tend to give birth at home) or the richest (who tend to use private hospitals). We therefore cannot generalise our results with certainty to the richer and poorer groups in the region, but we would expect the trend to be similar. Loss to follow‐up of families from urban locations and mothers with high levels of education may have influenced the results. The differences, however, were small and we do not believe would have changed the conclusions.

In summary, our findings suggest that SES at birth affects the growth of children, with maternal secondary education having the greatest positive effect. We suggest that interventions to improve SES prior to the birth of the child would help to improve growth and would be of long‐term benefit.

## CONFLICTS OF INTEREST

The authors declare that they have no conflicts of interest.

## CONTRIBUTIONS

The study was designed by DD, DO, JCKW. Data collection was managed by DD; BPS, DSM, NS, AC provided management of the field site; DD, RMD analysed the data; DD, DK wrote the first draft. All authors were involved read and criticised drafts of the manuscript.

## Supporting information

Supplementary Table 1: Review of literature showing longitudinal studies that describe the effects of an asset‐based index of household wealth, land ownership or maternal education on child anthropometry and growth
