# Supplementary material for: Socioeconomic determinants of growth in a longitudinal study in Nepal
Source: Matern Child Nutr. 2017 Apr 27;14(1):e12462. doi: 10.1111/mcn.12462 (PMC5763270; doi:10.1111/mcn.12462)
Supplement: Supplementary file 1 — Supplementary Table 1: Review of literature showing longitudinal studies that describe the effects of an asset‐based index of household wealth, land ownership or maternal education on child anthropometry and growth [file MCN-14-e12462-s001.docx]

**Supporting information**

**Determinants of linear growth in a longitudinal study in southern Nepal**

Devakumar D, Kular D, Shrestha BP, Grijalva-Eternod C, Daniel RM, Saville NM, Manandhar DS, Costello A, Osrin D, Wells JCK

**Supplementary Table 1: Review of literature showing longitudinal studies that describe the effects of an asset-based index of household wealth, land ownership or maternal education on child anthropometry and growth**

| **Author and Journal** | **Marker of SES** | **Marker of SES** | **No. of participants and location** | **Outcome** | **Results** |
| --- | --- | --- | --- | --- | --- |
| Busert et al., (Busert et al., 2016) | Assets  Maternal education  (Busert et al., 2016) | PCA (toilet ownership, selected household assets (such as radio or electricity), land ownership and  income from a family member who had migrated for work)  Mother’s years of schooling (yrs) | Nepal  N=529  Aged 0-59 months  Followed up after 9 months and 29 months | HAD (conditional growth) | No significant association between wealth quintile and conditional growth in both growth periods (p<0.05)  No significant association between maternal schooling and conditional growth in both growth periods  9 months β coefficient -0.01 (-0.06, 0.04)  29 months β coefficient -0.05 (-0.10. 0.00) |
| Jones et al., (Jones et al., 2008) | Assets  Maternal education | PCA included maternal education (high school or less than high school), refrigerator and television ownership, water and toilet facilities to give SES index | South Africa  N=2293  Followed up birth to 1 year (n=450) and 2 years (n=401)  Philippines  N=2513  Followed up birth to 1 year (n=1820) and 2 years (n= 1710)  Both urban cohorts | Stunting | Philippines at 1 year of age  A lower SES index OR for stunting 0.61 (95% 0.53, 0.70)  Not owning a television OR for stunting 1.69 (95% CI 1.18, 1.87)  Not having indoor flush toilet OR for stunting 1.49 (95% CI 1.18, 1.87)  Mother with less than high school education OR for stunting 1.42 (95% CI 1.08, 1.88)  Philippines at 2 years of age  A lower SES index OR for stunting 0.51 (95% CI 0.47, 0.58)  Not owning a television OR for stunting 1.72 (95% CI 1.27, 2.35)  Not having indoor flush toilet OR for stunting 1.72 (95% CI 1.38, 2.15)  Mother with less than high school education OR for stunting 2.04 (95% CI 1.56, 2.65)  South Africa  SES index or individual variable not significantly associated with stunting at 1 or 2 years  Mother with less than high school education was not significantly associated with stunting at 1 or 2 years. |
| Krishna et al., 2015  (Krishna et al., 2015) | Assets | Wealth index constructed from three equally weighted components- a housing quality index, a services quality index and a consumer durables index and ranges from 0 to 100. | Ethiopia, India  Peru, Vietnam  2 cohorts in each country  N= 10, 016  Two cohorts: a younger and an older  Younger cohort: Measurements taken at 6-18 months, 4-5 years, and 7-8 years  Older cohort Measurements taken at 7-8 years, 11-12 years and 14-15 years | HAZ and stunting | Analyses adjusted for clustering, child’s age, sex, caregiver’s educational attainment, household size, and place of residence (rural/urban).  Significant associations were seen in the younger cohort for Ethiopia, India and Vietnam and in the older cohort for Peru and Vietnam. |
| Lourenco et al., (Lourenco et al., 2012) | Assets  Land | Principal components analysis based on 14 appliances (tertiles) wealth index  Land ownership (yes or no) | Aged 0-10 years  n=256  Brazilian Amazon  Follow up after 4 and 6 years | HAZ | Those above the median wealth index compared to those below had a significantly higher HAZ score at age 5 years (β 0.30 (95% CI 0.06, 0.54) and 7 years β 0.25 (95% CI 0.02, 0.48), but not at <2 years and at 10 years  Land ownership significantly associated with HAZ at ages 5 years (β=0.31; 95% CI 0.01, 0.61), 7 years (β=0.33; 95% CI 0.06, 0.61) and 10 years, (β=0.34; 95% CI 0.05, 0.63) but not at <2 years |
| Mallard et al.(Mallard et al., 2014) | Assets  Maternal education | Principal components analysis (home ownership, sanitation facilities, floor type, connection to water, electricity, and telephone, number of meals per day, transport type and ownership of electrical appliances, animals and a vegetable garden)  Maternal education catergorised as primary school or less, secondary school and college/university | Age 6 months  n=811  Urban Zambia  Follow up 12 months | HAZ | Compared to lowest quintile at 18 months the middle quintile (β coefficient 0.21 (p=0.029)), fourth highest quintile (β coefficient 0.37 (p<0.001)) and highest quintile (β 0.34 p=0.001)) were significantly positively associated with HAZ.  Baseline maternal education effect on HAZ at 18 months, secondary school β 0.17 (p=0.023), college/university β 0.43 (p<0.001). |
| Matijasevich et al., (Matijasevich et al., 2012) | Maternal education | Mother’s schooling categorised as 0-4, 5-8 and > 9 complete school years | Pelotas, Brazil  Birth to 4 years  2106 Boys  1947 girls | Height (cm) | Adjusted model  Mothers schooling was significantly positively associated with boys growth at 0-3 months and 12-29 months (p<0.001). For girls, mother’s schooling was significantly associated with growth at 0-3 months and 12-32 months (p<0.001, p=0.001). |
| Patel et al., (Patel et al., 2011) | Maternal education | Maternal schooling placed into 3 categories.  Initial/incomplete/ common secondary, Advanced secondary/partial university and completed university | Belarus  N=11, 074  Birth to 6.5 years | Height, Leg length, trunk length (cm) | Comparing children of mothers who completed university with mothers who had initial/incomplete/common secondary education showed a difference of 0.71 cm (95% CI 0.36, 1.05) for standing height and 0.29 cm (95% CI 0.07, 0.51) for relative leg length.  Comparing children of mothers who had advanced secondary or partial university education with mothers who had initial/incomplete/common secondary education showed a difference of 0.44 cm (95% CI 0.26, 0.62) for standing height and 0.15 cm (95% CI 0.03, 0.27) for relative leg length. |
| Svefors et al., (Svefors et al., 2016) | Maternal education | No formal education, 1-5 years (primary school), more than 5 years of education | Rural Bangladesh  N=1054  Birth to 10 years | HAZ and stunting | In adjusted analysis (Model 2), children born by mothers with no education had the lowest HAZ scores -0.25 (95% CI -0.39 to -0.11) and the highest probability to be stunted at 10 years of age OR 1.74 (95% CI 1.17-2.81) as compared to children born by mothers with more than 5 years of education. |

Busert L.K., Neuman M., Rehfuess E.A., Dulal S., Harthan J., Chaube S.S., et al. (2016) Dietary Diversity Is Positively Associated with Deviation from Expected Height in Rural Nepal. *J Nutr* **146,** 1387-1393.

Jones L.L., Griffiths P.L., Adair L.S., Norris S.A., Richter L.M. & Cameron N. (2008) A comparison of the socio-economic determinants of growth retardation in South African and Filipino infants. *Public Health Nutr* **11,** 1220-1228.

Krishna A., Oh J., Lee J.K., Lee H.Y., Perkins J.M., Heo J., et al. (2015) Short-term and long-term associations between household wealth and physical growth: a cross-comparative analysis of children from four low- and middle-income countries. *Glob Health Action* **8,** 26523.

Lourenco B.H., Villamor E., Augusto R.A. & Cardoso M.A. (2012) Determinants of linear growth from infancy to school-aged years: a population-based follow-up study in urban Amazonian children. *BMC public health* **12,** 265.

Mallard S.R., Houghton L.A., Filteau S., Mullen A., Nieuwelink J., Chisenga M., et al. (2014) Dietary Diversity at 6 Months of Age Is Associated with Subsequent Growth and Mediates the Effect of Maternal Education on Infant Growth in Urban Zambia. *The Journal of Nutrition* **144,** 1818-1825.

Matijasevich A., Howe L.D., Tilling K., Santos I.S., Barros A.J. & Lawlor D.A. (2012) Maternal education inequalities in height growth rates in early childhood: 2004 Pelotas birth cohort study. *Paediatric and perinatal epidemiology* **26,** 236-249.

Patel R., Lawlor D.A., Kramer M.S., Davey Smith G., Bogdanovich N., Matush L., et al. (2011) Socioeconomic inequalities in height, leg length and trunk length among children aged 6.5 years and their parents from the Republic of Belarus: evidence from the Promotion of Breastfeeding Intervention Trial (PROBIT). *Ann Hum Biol* **38,** 592-602.

Svefors P., Rahman A., Ekstrom E.C., Khan A.I., Lindstrom E., Persson L.A., et al. (2016) Stunted at 10 Years. Linear Growth Trajectories and Stunting from Birth to Pre-Adolescence in a Rural Bangladeshi Cohort. *PloS one* **11,** e0149700.

**References**
